# Drug-likeness analysis of traditional Chinese medicines: 2. Characterization of scaffold architectures for drug-like compounds, non-drug-like compounds, and natural compounds from traditional Chinese medicines

**DOI:** 10.1186/1758-2946-5-5

**Published:** 2013-01-21

**Authors:** Sheng Tian, Youyong Li, Junmei Wang, Xiaojie Xu, Lei Xu, Xiaohong Wang, Lei Chen, Tingjun Hou

**Affiliations:** 1 Institute of Functional Nano & Soft Materials (FUNSOM) and Jiangsu Key Laboratory for Carbon-Based Functional Materials & Devices, Soochow University, Suzhou, Jiangsu, 215123, China; 2 College of Pharmaceutical Sciences, Soochow University, Suzhou, Jiangsu, 215123, China; 3 Department of Biochemistry, The University of Texas Southwestern Medical Center, 5323 Harry Hines Blvd., Dallas, TX, 75390, USA; 4 College of Chemistry and Molecular Engineering, Peking University, Beijing, 100871, China

**Keywords:** Scaffold, Drug-likeness, Traditional Chinese medicines, Murcko frameworks, Scaffold tree, Tree maps

## Abstract

**Background:**

In order to better understand the structural features of natural compounds from traditional Chinese medicines, the scaffold architectures of drug-like compounds in MACCS-II Drug Data Report (MDDR), non-drug-like compounds in Available Chemical Directory (ACD), and natural compounds in Traditional Chinese Medicine Compound Database (TCMCD) were explored and compared.

**Results:**

First, the different scaffolds were extracted from ACD, MDDR and TCMCD by using three scaffold representations, including Murcko frameworks, Scaffold Tree, and ring systems with different complexity and side chains. Then, by examining the accumulative frequency of the scaffolds in each dataset, we observed that the Level 1 scaffolds of the Scaffold Tree offer advantages over the other scaffold architectures to represent the scaffold diversity of the compound libraries. By comparing the similarity of the scaffold architectures presented in MDDR, ACD and TCMCD, structural overlaps were observed not only between MDDR and TCMCD but also between MDDR and ACD. Finally, Tree Maps were used to cluster the Level 1 scaffolds of the Scaffold Tree and visualize the scaffold space of the three datasets.

**Conclusion:**

The analysis of the scaffold architectures of MDDR, ACD and TCMCD shows that, on average, drug-like molecules in MDDR have the highest diversity while natural compounds in TCMCD have the highest complexity. According to the Tree Maps, it can be observed that the Level 1 scaffolds present in MDDR have higher diversity than those presented in TCMCD and ACD. However, some representative scaffolds in MDDR with high frequency show structural similarities to those in TCMCD and ACD, suggesting that some scaffolds in TCMCD and ACD may be potentially drug-like fragments for fragment-based and *de novo* drug design.

## Introduction

Natural products are generally considered as a rich source of biologically active substances [1]. Many drugs approved by the Food and Drug Administration (FDA) directly come from natural products. In the period of 1981–2002, 5% of the 1031 new chemical entities (NCE) approved as drugs by the FDA are natural products, and other 23% are natural-product-derived molecules [2]. Historically, 60% of cancer drugs and 75% of infectious disease drugs are derived from natural products [2]. Because natural products have been selected during evolution to bind to various proteins during their life-cycle, they are good starting points for drug discovery [3,4]. Traditional Chinese medicines (TCMs) are primarily based on a large number of herbal formulations that are used for the treatment of a wide variety of diseases. The discovery of hits or leads from natural compounds in TCMs has become a feasible and popular strategy in modern drug discovery pipelines [2].

With the rapid development of high-throughput screening (HTS) and combinatorial synthesis, it becomes possible to generate and evaluate tens of thousands of compounds in a very short period of time with relatively low cost. Unfortunately, the new drugs approved by the FDA did not soar in recent years and even declined slightly, and even only one *de novo* combinatorial compound was approved in the last 25 years before 2007 [5]. This low success rate may be partly caused by low chemotype, limited scaffold diversity and lack of biological relevant scaffolds of combinatorial compounds [6]. Therefore, searching and designing molecule collections with novel scaffolds and high structural diversity will offer more opportunities for molecules to become leads, and ultimately to become new drugs. It is believed that natural compounds are a good source of novel molecular scaffolds [2,5,7-9] and the scaffolds derived from natural compounds have preferable or privileged scaffold architectures [10]. Since the scaffolds of natural compounds are potentially valuable, how to characterize and define the scaffolds that are meaningful for drug design/discovery is the center question we are facing now. It is well known that ring systems form the cornerstone of molecules, and they determine the basic shapes and flexibilities of molecules [11]. In drug design process, ring systems are usually used as the core or central scaffolds to build virtual libraries, and the ring systems in known active compounds can usually be replaced or modified to find new active candidates by using the “scaffold hopping” technique [12].

**Figure 1:**
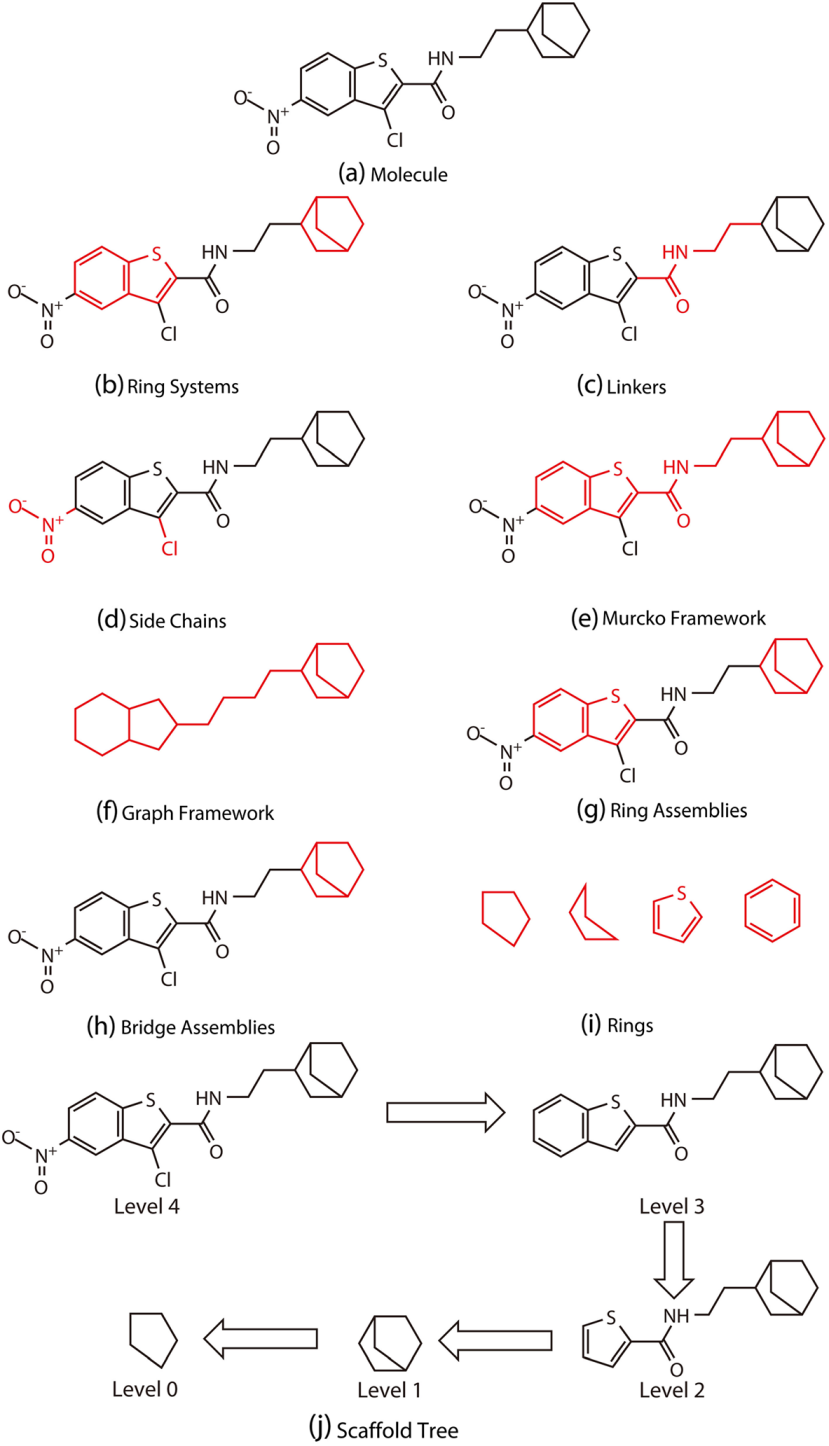
**A molecule depicted by different scaffold representations.** The molecule (**a**) is dissected into (**b**) Ring Systems: cycles within the graph representation of the molecules, (**c**) Linkers: atoms that are on the direct path connecting two ring systems, (**d**) Side Chains: any non-ring, non-linker atoms, and (**e**) Murcko Framework: the union of ring systems and linkers in a molecule; (**f**) Graph Framework can be obtained by considering all atoms and bonds in the molecule indentified as carbon and single bond. Different complexity-level ring systems include (**g**) Ring Assemblies: contiguous ring systems, (**h**) Bridge Assemblies: contiguous ring systems that share two or more bonds, and (**i**) Rings: individual rings; (**j**) The different levels of the Scaffold Tree.

To data, numerous approaches have been developed to analyze the scaffold architectures of different compound libraries [9,11,13-18]. In order to characterize the scaffold diversity of a compound library, a suitable representation or definition of scaffolds is required. In 1996, Bemis and Murcko proposed a method to dissect molecules in CMC into framework which is the union of ring systems and linkers in a molecule, side chains and linkers (Figure 1). The graph theory analysis shows that there were 1179 different frameworks present in 5120 known drugs and the 32 most frequently occurring frameworks accounted for 50% of the 5120 known drugs [13]. In 1999, Bemis and Murcko found that there were 1246 different side chains in CMC that have 5090 compounds and the average number of side chains per molecule is 4 [14].

In 2001, Lipkus proposed a simple strategy to organize chemical rings based on three integer descriptors, and he found that the distribution of 40,182 different ring topologies derived from a comprehensive collection of chemical rings from the CAS registry was not compact and had many significant voids [17].

In 2001, Lee et al. used a two-step protocol to determine whether natural products contain appealing novel scaffold architectures for potential use in combinatorial chemistry [9]. The ring systems were extracted from natural products and trade drugs and clustered on the basis of structural similarity in a Self-Organizing Map (SOM), which demonstrates that current trade drugs and natural products have several topological pharmacophore patterns in common. Approximately 35% of the ring systems in trade drugs were present in natural products, but only 17% of the ring systems found in natural products can be found in trade drugs.

In 2006, Ertl and coworkers investigated the simple aromatic ring systems present in a set of 149,437 bioactive compounds and only 780 unique simple aromatic scaffolds [11]. Additionally, 216 of these scaffolds were singletons (present only once in the entire bioactive collection). Moreover, only 10 such scaffolds are present in more than 1% of bioactive molecules and 64 in more than 0.1%. Self-organizing map (SOM) was then used to separate 780 scaffolds present in the bioactive molecules from 575,776 scaffolds present in a virtual library, and the results demonstrated that the 780 biologically active scaffolds are sparsely distributed in the chemical space, forming only a limited number of small, well-defined “activity islands”.

In 2005, Koch et al. developed a structural classification of natural products (SCONP), which arranges the scaffolds present in natural products (NP) in a tree-like fashion. The NP Scaffold Tree can be used as a strategic and guiding tool for the selection of underlying frameworks for NP-inspired compound library development [16]. Similarly, in 2007, Schuffenhauer et al. proposed the Scaffold Tree (ST) technique to give a hierarchical classification of chemical scaffolds obtained by pruning all terminal side chains [18]. The techique iteratively removes ring one by one according to a set of prioritization rules, and in the end the substructure with only one ring is obtained. The hierarchical structure for a molecule is numbered sequentially from Level 0 (one ring substructure) to Level *n* (the whole molecule) (Figure 1j), and Level *n*-1 represents the Murcko framework (Figure 1e). All-Level structures for a molecule are then combined into a tree. Recently, the Scaffold Tree technique has been widely used to analyze the scaffold diversity of compound libraries [19,20].

In 2010, we applied a brute force approach to enumerate all the possible scaffolds in a molecule for 1240 marketed drugs and 6932 drug candidates entering clinical trials. We found that the top 50 scaffolds cover about 52.6% of marketed drugs and 48.6% of drug candidates. The drug likeness of each scaffold was evaluated using the ratio of the hit rates in drug data set to that in the screening data set (a subset of the ZINC database containing 1.95 million entries) [21].

Apparently, many studies discussed above illustrate that many compound libraries do not have enough scaffold diversity. In previous studies, the scaffold diversity for natural products has been investigated [9,16]. However, to our knowledge, the study on the analysis of scaffold architectures and scaffolds for natural compounds from TCMs has never been reported. It is quite possible that the novel scaffolds present in natural compounds from TCMs may serve as good starting points for the development of natural product-like compound libraries. Here, the scaffold architectures and scaffold diversity were investigated for three classes of compounds collections, including MACCS-II Drug Data Report (MDDR), Available Chemical Directory (ACD), and Traditional Chinese Medicine Compound Database (TCMCD). The new version of the TCMCD database developed in our group contains 63,759 molecules identified from more than 5000 herbs used in traditional Chinese medicines (TCMs) [22]. To our knowledge, the number of molecules in TCMCD is the largest around the world. Here, we used three structural partition strategies to represent scaffolds, including (1) Murcko framework [13], (2) Scaffold Tree [18], and (3) a scheme based on rings, ring assemblies [23], bridge assemblies and side chains. Then, Tree Maps were used to visualize the distribution of molecular compounds over scaffolds based on the molecular fingerprint similarity of these scaffolds. The representative scaffolds in each dataset and the difference of the scaffold diversity between the studied datasets were highlighted by the Tree Maps.

## Methods and materials

### Datasets for scaffold analysis

The scaffold architectures of three datasets, including MDDR, ACD and TCMCD, were explored. The MDDR database was chosen as representatives for drug-like compounds, the ACD database as representatives for non-drug-like compounds, and the TCMCD database as representatives for natural compounds from TCMs. The TCMCD was developed in our group [22,24]. The latest version of TCMCD has 63,759 organic molecules identified from more than 5,000 herbs in TCMs. The protocol to preprocess the three datasets is described in the Additional file 1. It is well known that too large molecules usually do not have favorable absorption property, and therefore we set the cutoff for molecular weight to be 600 [25,26], and the sub-datasets, namely ACD1, MDDR1 and TCMCD1, respectively, were extracted by only choosing molecules with molecular weight less than 600. In total, there are 1,999,530, 123,927, 50,962 entries in ACD1, MDDR1 and TCMCD1, respectively.

Comparison study showed that the three studied datasets have different distributions of molecular weight [27]. In order to remove the influence of molecular size on scaffold analysis, we constructed two subsets of ACD1 and TCMCD1, which have similar molecular weight distribution to that of MDDR1. After applying all these preprocessing steps, we extracted a subset (ACD2) of 123,927 molecules from ACD1 and a subset (TCMCD2) of 33,961 molecules from TCMCD1, and both subsets have similar molecular weight (MW < 600) distributions to that of the MDDR subset (MDDR1). At last, we wanted to compare the structural differences among MDDR, ACD and TCMCD more equally and rationally, 33,961 molecules were extracted randomly from MDDR1 and ACD2, respectively, to construct the subsets MDDR2 and ACD3. The final three subsets, including TCMCD2, ACD3 and MDDR2, used for scaffolds analysis have the same number of molecules (33,961) and almost the same molecular weight distributions.

### Generation of scaffold architectures

The scaffolds for MDDR2, ACD3 and TCMCD2 were then generated by three scaffold representations. Here, Murcko frameworks developed by Bemis [13] and Scaffold Tree developed by Schuffenhauer [18], which are primarily used to characterize cyclic substructures of molecules, were used. Moreover, we also designed a simple protocol to identify scaffolds by checking the occurrence of cyclic substructures, including simple rings, ring assemblies, and bridge assemblies. Furthermore, the side chains attached to Murcko frameworks were also used in our analysis.

First, scaffolds were generated using the Scaffold Tree representation, which is a hierarchical classification of scaffolds shown in Figure 1j. First, Murcko frameworks of the studied compounds are generated, and they form the leaf nodes in hierarchical trees. By an iterative removal of rings according to predefined prioritization rules that favor the selection of non-scaffold-like fragments and retain the most functionalized ring systems, each molecule is chopped up into ever smaller pieces until the remaining fragment that only contains one ring substructure cannot be made even smaller. Then, for each molecule, we can get a list of ring systems at different levels of Scaffold Tree. The final single ring as the root node in the tree is named Level 0, and so forth, the subsequent levels or nodes in the tree are named numerically. Different molecules may have different levels, depending on the complexity or the number of rings in the studied molecules. Then the molecules with each scaffold are counted. The choice of the level for scaffold analysis is really arbitrary. The scaffolds at Level 0 are usually too simple to characterize the structural features of the studied molecules. When the level of a Scaffold Tree becomes higher, the scaffolds become more complicated and even identical to the Murcko framework. In addition, some compounds with low molecular weight or less molecular complexity usually do not have Level 3 or above. According to Langdon’s results [28], Level 1 of the Scaffold Tree is the best choice for the characterization of scaffold diversity. Here, considering the balance between molecular complexity and diversity for the molecules in the three studied datasets, Level 1 and Level 2 of the Scaffold Tree were used in our analysis. The Scaffold Tree for each dataset was generated by using the *linear fragmentation function* in the Molecular Operating Environment (MOE) suite [29]. An SVL (Scientific Vector Language) script was applied to the SDF file of each dataset. The Level 1 and Level 2 scaffolds were saved for the further analysis.

Murcko frameworks (Figure 1e), rings (Figure 1i), ring assemblies (Figure 1g), and bridge assemblies (Figure 1h), were generated using the *Generate Fragments* component in Pipeline Pilot 7.5. Because side chains of some molecules are also valuable for a variety of purposes, for example, improving synthetic accessibility, solubility and reducing metabolism and toxicity of the studied molecules [14], we generated the side chains of the studied datasets. The arrays of side chains were generated using the *Generate Fragments* component in Pipeline Pilot 7.5.

### Scaffold diversity analysis

The scaffold diversity analysis was performed on the TCMCD2, ACD3 and MDDR2 datasets. For comparing the scaffold diversity of the studied datasets, duplicated ring systems and side chains were removed, and the unique ring systems and side chains were obtained. The scaffold diversity for each dataset was characterized by two types of diversity measurements: the distribution of molecules over the unique scaffolds present in the dataset and the structural diversity of the scaffolds. In the current work, the scaffold counts and the cumulative frequencies of scaffolds are used to measure the distribution of molecules over the unique scaffolds present in the dataset, and the Tree Maps can characterize both the distribution and structural diversity of the scaffolds.

The number of each scaffold architecture, also represented as scaffold frequency, was counted for Murcko frameworks, the Level 1 and Level 2 scaffolds, rings, ring assemblies, bridge assemblies, and side chains in each dataset. Then, the scaffolds were sorted by the scaffold frequency from most to least frequent. Finally, the pencentage of the cumulative scaffold frequency (CSF) for each dataset was plotted [28].

### Generation of Tree Maps

Unlike the traditional approach to represent tree structures by a directed graph with the root node at the top and children nodes below the parent node with lines connecting them, Tree Maps proposed by Shneiderman use a 2D space-filling approach and use circles or rectangles to represent designated properties of molecules for clearly intuitive visualization [30]. Tree maps have been used to visualize hierarchical clustering by organizing molecular data on the basis of the similarity between chemical structures or similarity across a predefined profile of biological assay values and to prepare visual representations of molecular structure hierarchies alongside activity information.

Here, we used tree maps to analyze the structural diversity of different scaffold architectures by using the TreeMap software [31]. The scaffold frequency of the scaffolds can be represented by the color and area of the circles. The Tree Maps can highlight both scaffold structural diversity and the distribution of compounds over scaffolds. First, the Level 1 scaffolds of MDDR, ACD and TCMCD were clustered using ECFP_6 fingerprints [32]. The reason for using ECFP_6 fingerprints is that the structural difference between MDDR and ACD based on ECFP_6 is more obvious than that based on the other fingerprints according to our analysis. The *Cluster Molecules* component in Pipeline Pilot was used to cluster the scaffold architectures of three databases based on ECFP_6, and the average number of compounds *per* cluster was set to 50. This protocol randomly selects a molecule from the data set as the first cluster center and then selects the remaining cluster centers to achieve maximum dissimilarity to the first cluster center and each other. After the cluster center molecules are assigned, the ownership of each remaining molecule to which cluster is then determined based on their similarity to the center molecule. The method is order dependent, because the randomly selected molecules are dependent on the order of the molecules entering the component. However, Langdon and co-workers pointed out that the order dependency of the *Cluster Molecules* component did not have major effect on the clustering results used to visualize the molecular data sets in the Tree Maps [28]. Therefore, we clustered the scaffolds for each dataset without repeating the clustering procedure. After clustering the scaffolds, each scaffold had a cluster number (1, 2, 3, et al.) to represent the cluster that the scaffold belongs to, a cluster center number (1 or 0) to represent if this scaffold is a cluster center or not, a cluster size to represent the total number of the scaffolds that belong to the same cluster, and the value of DistanceToClosest that is the Tanimoto distance between each scaffold and the cluster center scaffold in the same cluster.

## Results and discussions

### The analysis of scaffold counts

As discussed above, Murcko frameworks, ring assemblies, bridge assemblies, rings, Scaffold Tree can only characterize molecules with ring systems. For MDDR, ACD and TCMCD, the percentage of the molecules which do not have any ring system are 1.19%, 1.84%, and 3.14%, respectively, indicating that the vast majority of molecules in the three datasets have ring systems. At the same time, we observed that the percentage of the molecules that have Level 1 scaffolds for MDDR, ACD and TCMCD is 93.58%, 88.05% and 90.46%, respectively, and the percentage of the molecules that have Level 2 scaffolds for MDDR, ACD and TCMCD is 77.61%, 60.31% and 74.05%, respectively. But the percentage of molecules that have Level 3 or even higher level scaffolds is less than 50%. So, using Level 2 or Level 3 scaffolds to characterize the structural features of MDDR, ACD and TCMCD and to compare the scaffold diversity among them are appropriate and reasonable.

The numbers of the scaffolds at the different levels of the Scaffold Tree for MDDR, ACD and TCMCD are listed in Table 1. Moreover, the numbers of the different ring systems, including Murcko frameworks, ring assemblies, bridge assemblies and rings, and the number of the side chains of MDDR, ACD and TCMCD are summarized in Table 2. Because the studied datasets have almost the same molecular weight distribution and the same number of molecules, the influence of molecular weight on structural analysis can be effectively removed.

**Table 1:** The number of the scaffolds at the different levels of the Scaffold Tree for MDDR, ACD and TCMCD

**Level**	**No. of Scaffolds**			**No. of non-duplicated Scaffolds**		
	**MDDR**	**ACD**	**TCMCD**	**MDDR**	**ACD**	**TCMCD**
Level 0	33558	33336	32893	1232	673	1047
Level 1	31780	29902	30722	6386	4588	4053
Level 2	26358	20483	25149	13840	7334	6351
Level 3	15749	11928	16848	11117	5641	5810
Level 4	5848	4544	8521	4686	2778	3866
Level 5	1250	1134	2753	1046	752	1563
Level 6	212	171	665	170	131	426
Level 7	53	39	165	26	37	114
Level 8	12	16	20	12	16	18
Level 9	0	5	13	0	5	11
Level 10	0	2	7	0	2	5
Level 11	0	0	3	0	0	3
Level 12	0	0	3	0	0	3
Level 13	0	0	1	0	0	1

**Table 2:** The number of fragments with Murcko frameworks, ring assemblies, rings, bridge assemblies and side chains present in MDDR, ACD and TCMCD

**Scaffold architecture**	**No. of Scaffolds**			**No. of non-duplicated Scaffolds**		
	**MDDR**	**ACD**	**TCMCD**	**MDDR**	**ACD**	**TCMCD**
Murcko frameworks	33568	33341	32926	21172	13029	10786
Ring assemblies	85904	82352	54742	3394	1568	5957
Rings	101978	115972	121665	484	764	614
Bridge assemblies	387	1538	5381	114	245	971
Side Chains	460178	431939	445889	8735	6332	4897

As shown in Table 2, the numbers of the Murcko frameworks for MDDR, ACD and TCMCD are 33,568, 33,341, and 32,926, respectively, which demonstrate that most molecules in the studied datasets contain ring systems. However, the numbers of the non-duplicated Murcko frameworks are 21,172, 13,029, and 10,786, respectively. As shown in Figure 1, Murcko frameworks are the scaffold architectures by dissecting the side chains (Figure 1d) to get the union of ring systems (Figure 1b) and linkers (Figure 1c) of the studied molecules, and therefore they can be considered as the specific molecular structural signature. The significant large number of the Murcko frameworks present in MDDR clearly indicates that the diversity of the Murcko frameworks for MDDR is the highest (Table 2). Moreover, the number of the non-duplicated side chains present in MDDR (8735) is also larger than those present in ACD (6332) and TCMCD (4897). On average, the diversity of the molecules in MDDR is highest, which is well consistent with the results reported by Langdon [28]. Langdon and coworkers found that the approved drugs in Drugbank have more scaffold diversity than the other compound collections.

The rings (Figure 1i) generated by dissecting all of conjugated ring and bridged ring systems of the entire molecule usually represent simple ring systems (three-membered, four-membered rings, et al.). The non-duplicated numbers of the simple ring scaffolds for MDDR, ACD and TCMCD are 484, 764 and 614, respectively, indicating that ACD has more unique simple ring systems. As shown in Table 2, the number of the unique ring assemblies for TCMCD is 5957, which is obviously larger than that for MDDR (3394) and substantially larger than that for ACD (1568). Ring assemblies are defined as the remaining fragments when all non-ring bonds are removed, and they can be used to characterize the complicated ring systems present in molecules. Therefore, it is obvious that the TCMCD has more complicated ring systems. Furthermore, the number of the bridge assemblies for TCMCD (971) is substantially larger than those for MDDR (114) and ACD (245). According to the definition shown in Figure 1, bridge assemblies belong to ring assemblies, and they can characterize any rings that share more than one bond in common. Therefore, it is predicted that molecules in TCMCD contain more conjugated ring systems rather than bridged ring systems, since TCMCD has more non-duplicated ring assemblies than ACD and MDDR. In summary, the molecules in TCMCD have more complicated ring systems than those in MDDR and ACD.

**Figure 2:**
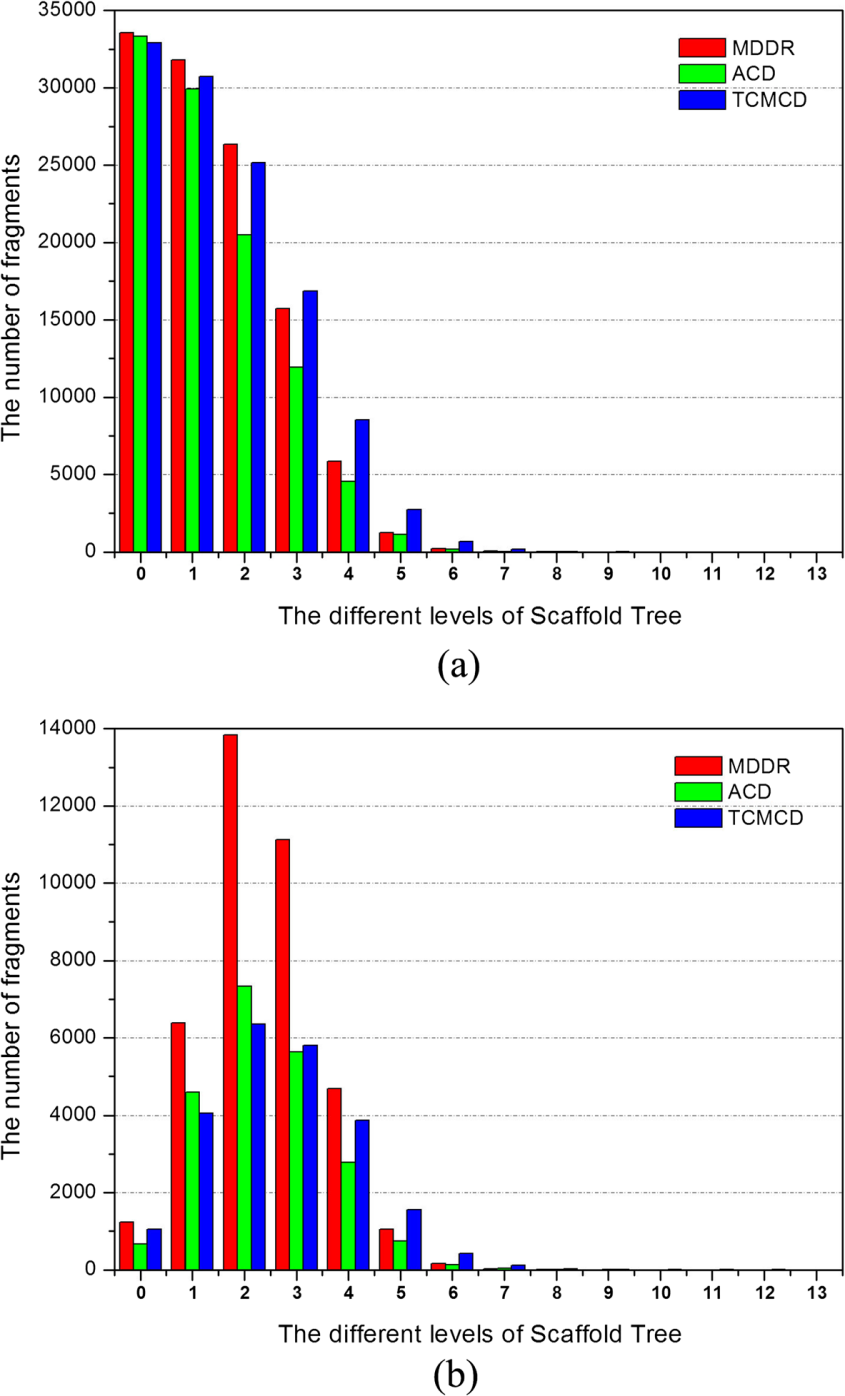
(a) The numbers of the scaffolds at different levels of the Scaffold Tree for MDDR, ACD and TCMCD; (b) The numbers of the non-duplicated scaffolds at different levels of the Scaffold Tree for MDDR, ACD and TCMCD.

Then, we analyzed the scaffolds at different levels of the Scaffold Tree for the studied datasets (Table 1 and Figure 2). The numbers of the Level 1 scaffolds for MDDR, ACD and TCMCD are 31,780, 29,902 and 30,722, respectively, and those of the Level 2 scaffolds are 26,358, 20,483 and 25,149, respectively. With the increase of the level of Scaffold Tree, the numbers of the scaffolds for the studied datasets decrease rapidly. When the level of the Scaffold Tree increases up to Level 6, the numbers of the scaffolds for MDDR, ACD and TCMCD are 212, 171 and 665, respectively. Moreover, the scaffolds cannot be found in MDDR when the level of the Scaffold Tree increases to Level 9. As shown in Table 1, most molecules in three studied datasets contain the scaffolds with two or more rings connected by the linkers. According to the numbers of the non-duplicated scaffolds at different levels of the Scaffold Tree, we found that MDDR has the largest number of the non-duplicated scaffolds from Level 0 to Level 4. Especially, the numbers of the scaffolds at Level 2 and Level 3 in MDDR are 13,840 and 11,117, respectively, which are about twice as many as those in ACD and TCMCD. However, when the level of Scaffold Tree increases further to Level 5 and higher, the number of the non-duplicated scaffolds in TCMCD becomes the largest.

Our observations show that the diversity of simpler ring systems (from Level 0 to Level 4) with one to five rings in MDDR is the highest, but that of more complicated ring systems in TCMCD is the highest. As we discussed above, based on the number of the non-duplicated Murcko frameworks, the molecules in MDDR have the highest diversity. Now, we also know that from Level 0 to Level 4 especially at the Level 2 and Level 3 scaffolds in MDDR are more diverse than those in ACD and TCMCD. Therefore, we can make the following conclusion: the highest diversity of MDDR might be determined by the high complexity and diversity of linkers (Figure 1c) between the fragments at lower levels of Scaffold Tree (three ring systems at Level 2 or four ring systems at Level 3). Finally, as shown in Figure 2a, one interesting phenomenon was observed: at almost all different levels of Scaffold Tree, the number of the unique scaffolds in MDDR is closer to that in TCMCD rather than that in ACD, suggesting that, on the whole, MDDR is more similar to TCMCD than ACD.

### Cumulative frequencies of the Murcko frameworks and the scaffolds at Levels 1 and 2 of the Scaffold Tree

As shown in Table 1, more than 88% of the studied compounds have two-ring systems at Level 1 and more than 60% of the studied compounds have three-ring systems at Level 2. Therefore, the Level 1 and Level 2 scaffolds can represent the whole structural features of the studied molecules.

**Figure 3:**
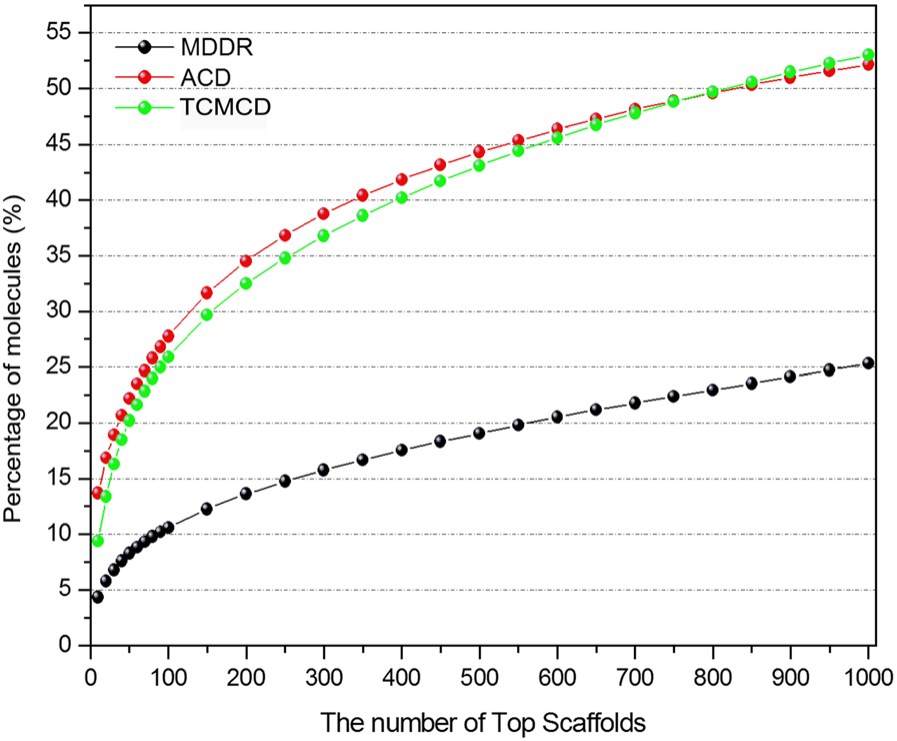
The percentage of the molecules that contain the top 1000 Murcko frameworks for MDDR, ACD and TCMCD.

**Figure 4:**
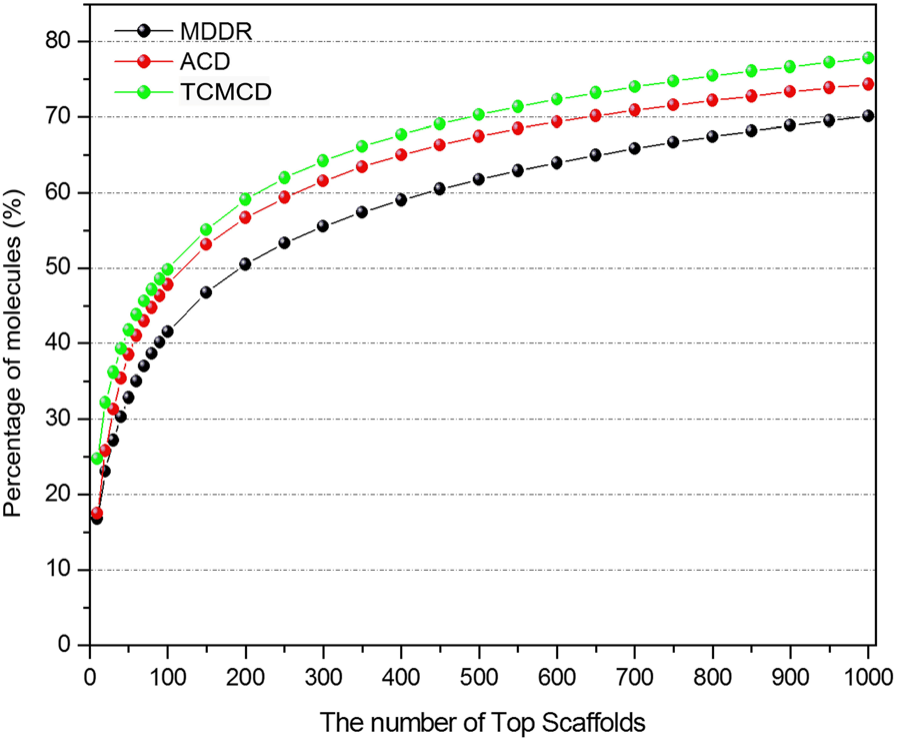
The percentage of the molecules that contain the top 1000 Level 1 scaffolds of the Scaffold Tree for MDDR, ACD and TCMCD.

**Figure 5:**
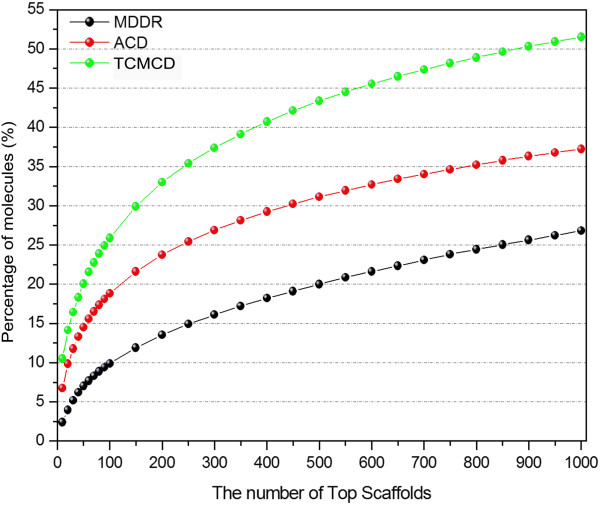
The percentage of the molecules that contain the top 1000 Level 2 scaffolds of the Scaffold Tree for MDDR, ACD and TCMCD.

Here, the cumulative frequencies of the molecules with the Murcko frameworks, scaffolds at Levels 1 and 2 were computed. By sorting the frequencies of the scaffolds, the top 1,000 scaffolds for the Murcko frameworks, Level 1 or Level 2 were obtained. Then, the cumulative frequencies of the molecules with the top 1000 scaffolds in MDDR, ACD and TCMCD for Murcko frameworks, Level 1 and Level 2 are displayed in Figures 3, 4, and 5. As shown in Figure 3, the curves of the accumulative frequencies of the Murcko frameworks for TCMCD and ACD are steeper than those for MDDR, indicating that the most frequently occurring Murcko frameworks in TCMCD and ACD represent more molecules than those in MDDR. For example, the top 100 most frequently occurring Murcko frameworks can be found in about 25.91% of molecules in TCMCD and 27.76% of molecules in ACD, but only about 10.57% of molecules in MDDR. For the top 1000 most frequently occurring Murcko frameworks, 53%, 52.17% and 25.32% of molecules in TCMCD, ACD and MDDR can be represented. Our results suggest that more than half of the molecules in TCMCD and ACD can be represented by the top 1000 Murcko frameworks, but just about a quarter of the molecules in MDDR can be represented by the top 1000 Murcko assemblies. As we mentioned above, the numbers of the unique Murcko frameworks in MDDR, ACD and TCMCD are 21,172, 13,029, and 10,786, respectively (Table 2). The cumulative scaffold frequency (CSF) plots for the Mucko framework representation further confirm that, in general, the molecules in MDDR have higher diversity than those in TCMCD and ACD.

The CSF plots for Level 1 and Level 2 scaffolds are displayed in Figures 4 and 5. The tendency of the CSF curve for Level 2 is similar to that for the Murcko frameworks shown in Figure 5. With the increased number of the Level 2 scaffolds, the CSFs of the molecules in TCMCD and ACD increase more rapidly than those in MDDR. Around 25.87%, 18.81% and 9.88% of molecules in TCMCD, ACD and MDDR can be represented by the top 100 Level 2 scaffolds. And around 51.51%, 37.22% and 26.8% of molecules in TCMCD, ACD and MDDR can be represented by the top 1,000 Level 2 scaffolds.

However, for the Level 1 scaffold, the CSF curves for the three studied datasets increase much more steeply (Figure 4) than those for the Murcko frameworks and Level 2 scaffolds. About 24.71%, 17.48% and 16.78% of molecules in TCMCD, ACD and MDDR can be represented by the top 10 most frequent Level 1 scaffold architectures, and about 77.85%, 74.31% and 70.12% of molecules in TCMCD, ACD and MDDR can be represented by the top 1000 Level 1 scaffold architectures. In summary, more than 88% of molecules in MDDR, ACD and TCMCD contain the Level 1 scaffolds and more than 70% of molecules in MDDR, ACD and TCMCD can be represented by the top 1000 most frequently occurring Level 1 scaffolds. The top 20 most frequently occurring Level 1 scaffolds among MDDR, ACD and TCMCD are shown in Additional file 1: Figure S1. Therefore, using the Level 1 scaffolds to evaluate the structural diversity and visualize distribution of molecules over scaffolds by Tree Maps are reasonable.

### The Similarity among MDDR, ACD and TCMCD

In the structural scaffolds used for the analysis, Murcko frameworks can be used to represent the overall features of the studied molecules and the Level 1 scaffolds can be used to characterize the core ring systems of the studied molecules. The results shown in the previous two sections indicate that the numbers of the Murcko frameworks and Level 1 scaffolds for MDDR, ACD and TCMCD are different. Here, based on ECFP_6 fingerprints, we evaluated the similarity of the Murcko frameworks and Level 1 scaffolds among MDDR, ACD and TCMCD. The numbers of the similar Murcko frameworks and level 1 scaffolds based on different cutoffs of similarity are listed in Tables 3 and 4.

**Table 3:** The number of the Murcko frameworks in one dataset that are similar to those in another dataset based on different similarity cutoff of ECFP_6 fingerprint

**Similarity**	**MDDR in ACD**	**MDDR in TCMCD**	**ACD in MDDR**	**ACD in TCMCD**	**TCMCD in MDDR**	**TCMCD in ACD**
=1	1191	570	1210	663	788	1141
≥0.9	1227	590	1258	674	812	1170
≥0.8	1316	644	1363	710	875	1244
≥0.7	1638	769	1620	791	989	1382
≥0.6	2601	1154	2448	1057	1277	1803
≥0.5	5310	2348	4568	1917	2157	2880
≥0.4	10982	5434	8187	4150	4047	4767
≥0.3	17914	12253	11753	8335	6968	7179
≥0.2	20923	19731	12953	12523	9713	9679
≥0.1	21172	21172	13029	13027	10786	10784
≥0	21172	21172	13029	13029	10786	10786

**Table 4:** The number of the Level 1 scaffolds in one dataset that are similar to those in another dataset based on different similarity cutoff of ECFP_6 fingerprint

**Similarity**	**MDDR in ACD**	**MDDR in TCMCD**	**ACD in MDDR**	**ACD in TCMCD**	**TCMCD in MDDR**	**TCMCD in ACD**
=1	1319	645	1306	537	715	646
≥0.9	1339	654	1341	542	728	655
≥0.8	1393	679	1405	555	767	697
≥0.7	1525	750	1496	589	872	792
≥0.6	1834	928	1783	697	1016	926
≥0.5	2605	1384	2468	1100	1307	1206
≥0.4	3920	2546	3494	2039	1835	1776
≥0.3	5353	4333	4255	3368	2770	2685
≥0.2	6215	5939	4541	4349	3800	3693
≥0.1	6386	6386	4588	4587	4053	4053
≥0	6386	6386	4588	4588	4053	4053

**Figure 6:**
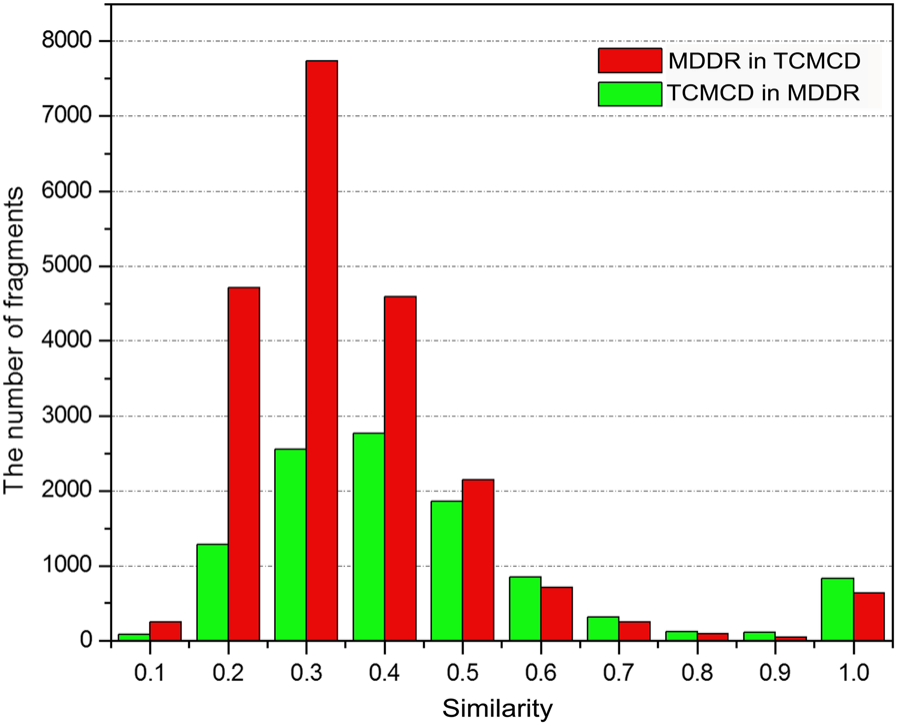
The number of the Murcko frameworks in MDDR that are similar to those in TCMCD based on different minimum similarity of ECFP_6 fingerprint.

As shown in Table 3, when the cutoff was set to the similarity higher than 0.5, the number of the Murcko frameworks in MDDR that are similar to those in ACD is 5310, and that of the Murcko frameworks in ACD that are similar to those in MDDR is 4568 (Figure 6). Therefore, about 25.1% (5310/21,172) of the Murcko frameworks in MDDR have similar Murcko frameworks in ACD, and 35.1% (4568/13,029) of the Murcko frameworks in ACD have similar Murcko frameworks in MDDR. The similar phenomenon could also be observed for the Level 1 scaffolds in MDDR and ACD (Table 4). About 40.8% (2605/6386) of the Level 1 scaffolds in MDDR has similar Level 1 scaffolds in ACD, and 53.8% (2468/4588) of the Level 1 scaffolds in ACD has similar Level 1 scaffolds in MDDR.

It is well-known that MDDR and ACD are traditionally considered as drug-like and non-drug-like compound collections. However, according to our analysis, we still found obvious scaffold overlaps between them. At this point, some readers may raise this question: do the ring systems (Murcko frameworks and Level 1 scaffolds) of ACD can be served as promising core ring substructures for drug design/discovery? Considering the high overlaps of the scaffolds between MDDR and ACD, it is quite possible that some ring systems in ACD might provide some promising substructures for drug discovery.

**Figure 7:**
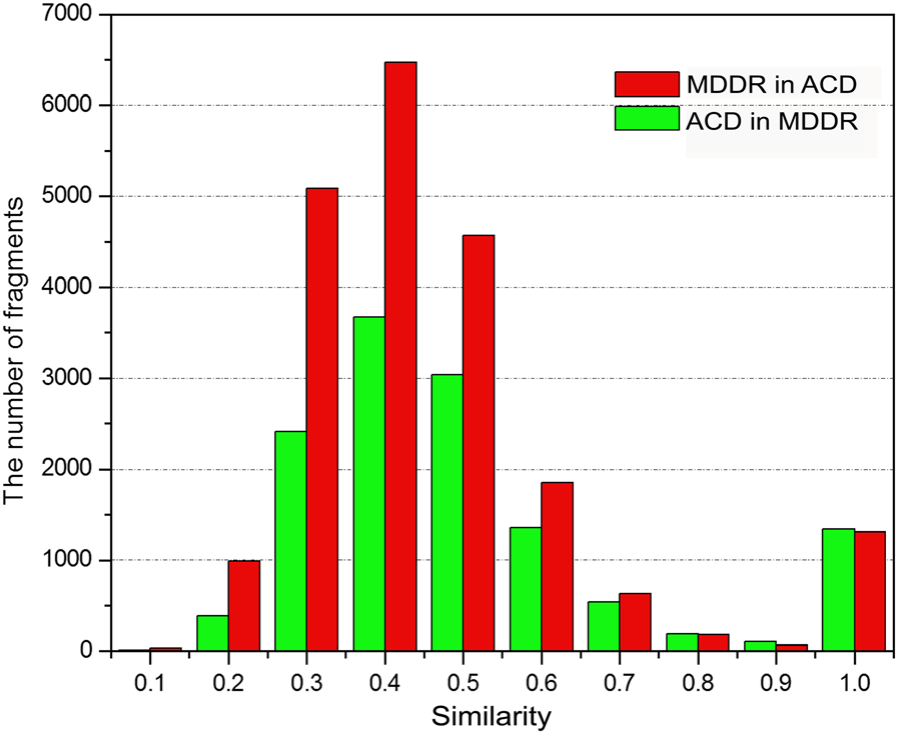
The number of the Murcko frameworks in MDDR that are similar to those in ACD based on different minimum similarity of ECFP_6 fingerprint.

**Figure 8:**
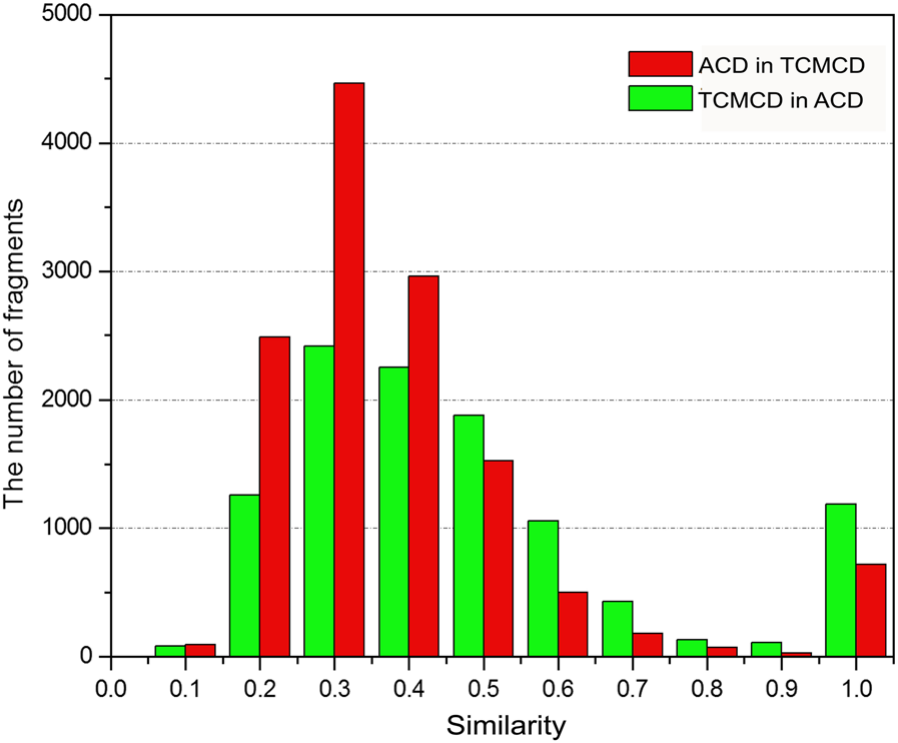
The number of the Murcko frameworks in ACD that are similar to those in TCMCD based on different minimum similarity of ECFP_6 fingerprint.

As shown in Table 3, the number of the Murcko frameworks in MDDR that are similar to those in TCMCD is 2348, when similarity of 0.5 was used as the cutoff. Using the same cutoff, the number of the Murcko frameworks in TCMCD that are similar to those in MDDR is 2157 (Figure 7). Similarly, when using the cutoff of similarity higher than 0.5, the number of the Level 1 scaffolds in MDDR to those in TCMCD is 1384. And the number of the Level 1 scaffolds in TCMCD to those in MDDR is 1307 (Table 4). That is to say, by setting the cutoff of similarity higher than 0.5, about 20.0% (2157/10,786) of the Murcko frameworks in TCMCD has similar Murcko frameworks in MDDR, while only 11.1% (2348/21,172) of the Murcko frameworks in MDDR has similar Murcko frameworks in TCMCD; about 32.2% (1307/4,053) of the Level 1 scaffolds in TCMCD has similar Level 1 scaffolds in MDDR, while only 21.7% (1384/6,386) of the Level 1 scaffolds in MDDR has similar Level 1 scaffolds in TCMCD. This observation is not surprising because according to the discussions shown above the scaffolds in MDDR have the highest diversity. It is obvious that there are structural overlaps of scaffolds between MDDR and TCMCD, but TCMCD still contains many novel ring systems (Murcko frameworks and Level 1 scaffolds) that cannot be found in MDDR. Moreover, when Level 1 scaffolds are compared, the percentage of MDDR scaffolds similar to those in TCMCD (1,384/6386 = 21.7%) is obviously lower than the percentage of MDDR scaffolds similar to those in ACD (2605/6386 = 40.8%). In addition, the percentage of ACD Level 1 scaffolds similar to those in TCMCD (1100/4588 = 24.0%) is obviously lower than the percentage of ACD scaffolds similar to those in MDDR (2468/4588 = 53.8%), suggesting that some Level 1 scaffolds in TCMCD are quite novel, and they are different from those in ACD and MDDR. Besides, similar conclusion can also be obtained by analyzing the similarity of the Murcko frameworks among the three datasets (Table 3 and Figures 6, 7, and 8). We believe that these novel ring systems (Murcko framework and Level 1 scaffolds) in TCMCD may be very potential for fragment-based drug design.

### Tree Maps

In the previous sections, the scaffold diversity of MDDR, ACD, and TCMCD was evaluated based on the distribution of molecules over scaffolds; moreover, the similarity of the scaffolds among MDDR, ACD and TCMCD was also examined. However, we know little about the distribution and structural diversity of the scaffolds. In order to answer this question, Tree Maps was used to visualize the structural diversity of the scaffolds within the overall dataset.

**Figure 9:**
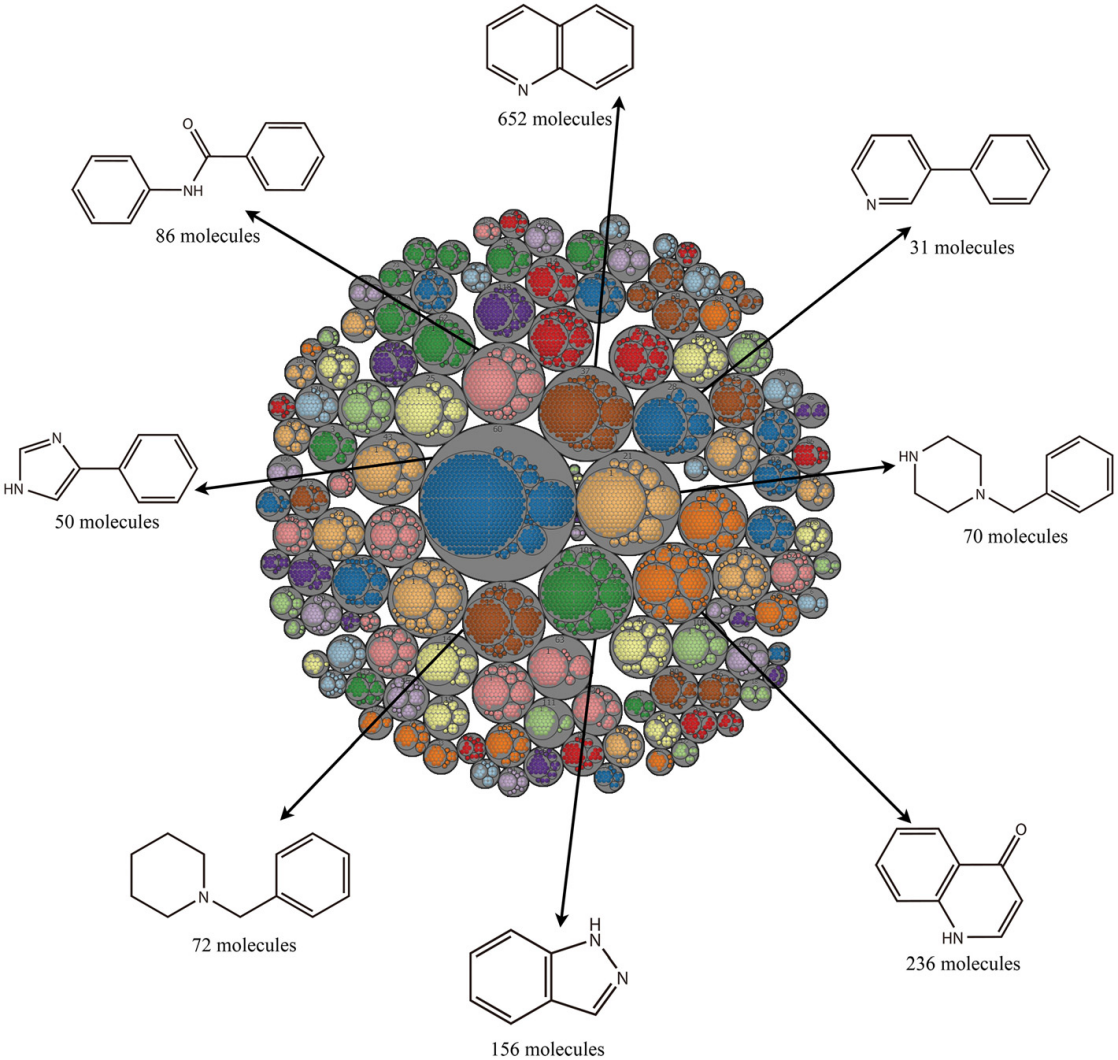
**Tree Map for the Level 1 scaffolds of MDDR.** Scaffolds are represented by different colored circles, and similar scaffolds are clustered in the independent gray circles. The most frequently occurring scaffolds for the eight largest clusters are depicted.

**Figure 10:**
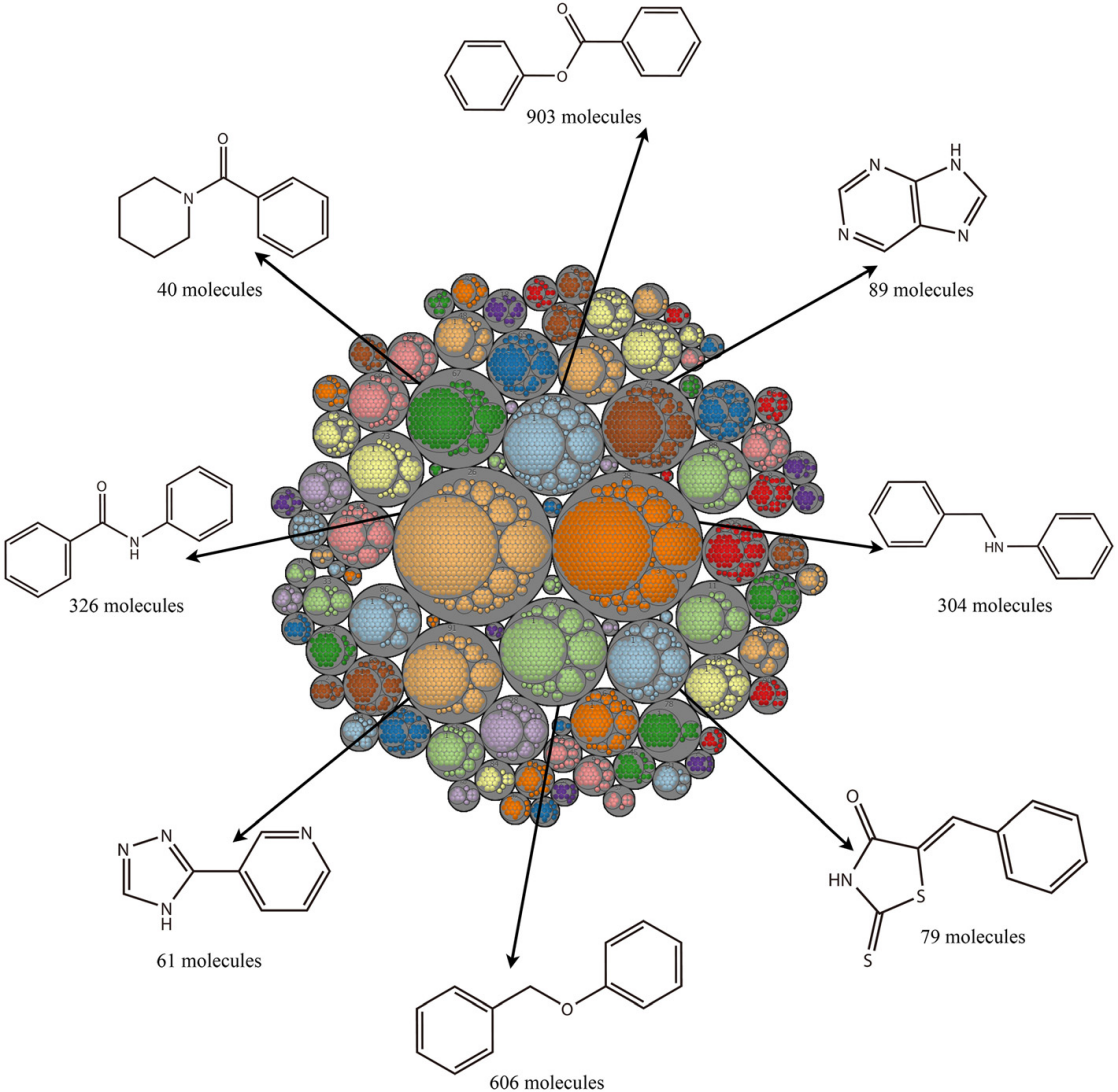
**Tree Map for the Level 1 scaffolds of ACD.** The most frequently occurring scaffolds for the eight largest clusters are depicted.

**Figure 11:**
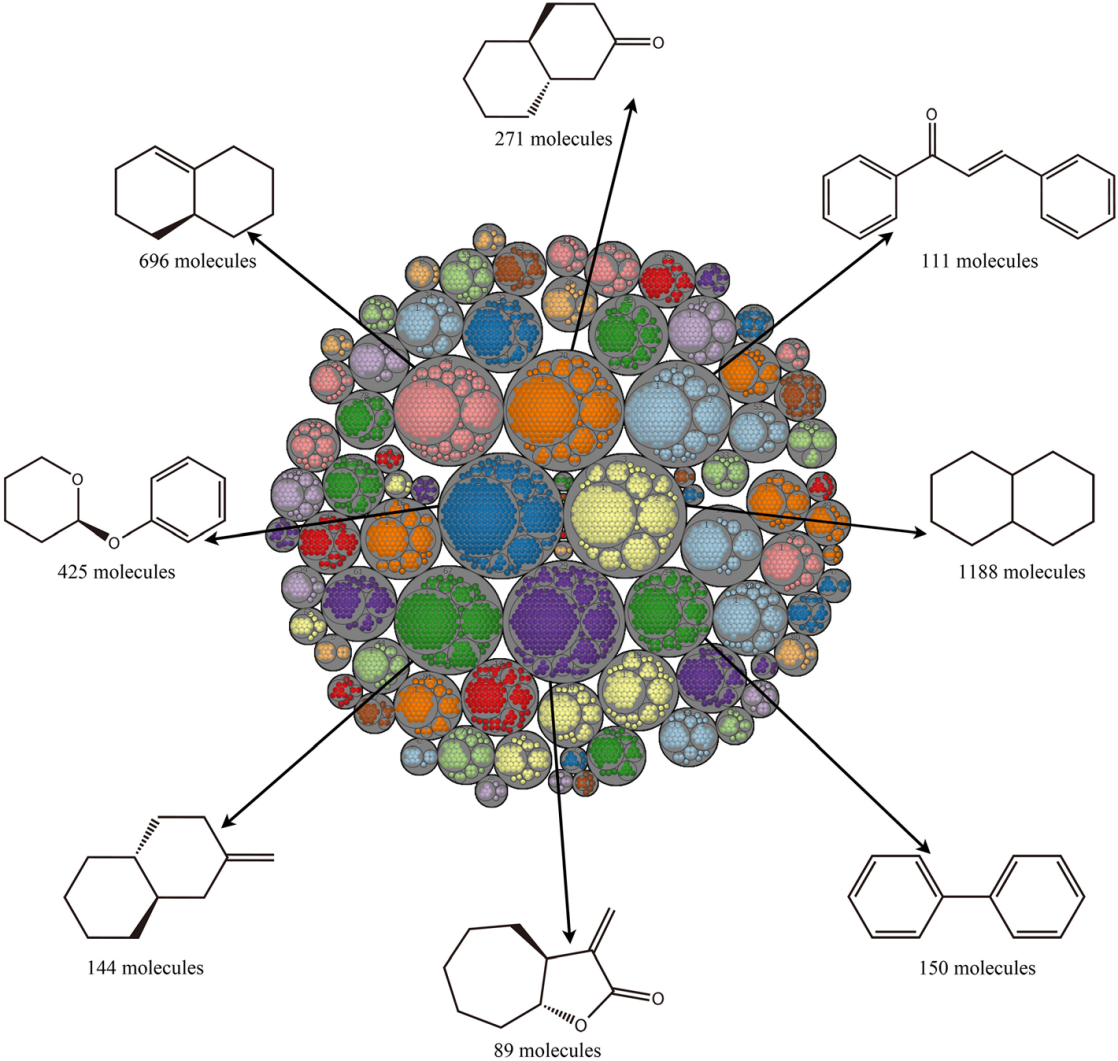
**Tree Map for the Level 1 scaffolds of TCMCD.** The most frequently occurring scaffolds for the eight largest clusters are depicted.

The Level 1 scaffolds for each dataset were clustered by their fingerprint similarity using the ECFP_6 fingerprints. The Tree Maps of MDDR, ACD and TCMCD are depicted in Figures 9, 10, and 11. Each gray circle represents an independent cluster. The size (big or small circle with same color) of the circles in each gray circle is proportional to the number of scaffold architectures with same frequency. The largest circle in each clustering group (gray circle) has the largest number of scaffolds. The 2-D structures and the number of the scaffolds for the eight largest groups with the highest frequency in MDDR, ACD and TCMCD are depicted in the Tree Maps. According to our analysis, the numbers of the clusters in MDDR, ACD and TCMCD are 128, 92 and 82, respectively. The comparison between Figures 9, 10 and 11 indicates that the Tree Maps for MDDR have more clusters highlighted by gray circles. That is to say, the Level 1 scaffolds in MDDR have more sparse distributions than those in ACD and TCMCD, indicating that the Level 1 scaffolds in MDDR have higher structural diversity than those in TCMCD and ACD.

Finally, we found that the most frequently occurring scaffolds of the clusters for ACD, MDDR and TCMCD were different in some extent. For exam-ple, as shown in Figure 11, the scaffold, (3aS,8aR)-3-methylene-octahydrocyclohepta[b]furan-2-one, is more complicated than the other scaffolds show in Figures 9 and 10. Certainly, the similarity between the Level 1 scaffold architectures in MDDR and those in ACD can also be observed. For example, the ring system with the highest frequency in the biggest gray circle for ACD is N-phenylbenzamide (Figure 10), which is the same to one of the most frequently occurring Level 1 scaffold in MDDR shown in Figure 9. In addition, the ring systems in MDDR with the highest frequency in the biggest gray circles is 4-phenyl-1H-imidazole, which is quite similar to one of the most frequent ring system found in ACD, 3-(4H-1,2,4-triazol-3-yl)pyridine. Moreover, as shown in Figures 9 and 11, one most frequently occurring scaffold found in 236 molecules in MDDR (Figure 9) is similar to some of the most frequently occurring scaffolds in TCMCD shown in Figure 11. Based on our observations, we believe that the scaffolds extracted from TCMCD can give valuable guidance to develop new leads or drugs.

## Conclusions

We have used different scaffold representations to examine the structural diversity of MDDR, ACD and TCMCD. Our analysis shows that the number of the unique Murcko frameworks for MDDR is much larger than those for ACD and TCMCD. At the same time, MDDR has the largest number of the non-duplicated side chains. Therefore, we believe that the molecular diversity of MDDR is higher than those of ACD and TCMCD.

The analysis of the ring assemblies and bridge assemblies suggests that natural compounds in TCMCD are more complicated than molecules in ACD and MDDR. By analyzing the different levels of the Scaffold Tree for the three datasets, we found that at the lower levels (from Level 0 to 4) the scaffold diversity for MDDR is the highest, while at the higher Levels (Level 5 or even higher) the scaffold diversity for TCMCD is the highest. Consequently, we realized that the higher molecular diversity of MDDR may be explained by the complexity and diversity of the linkers to connect the lower level ring systems.

By analyzing the cumulative frequency of the Murcko frameworks, the Level 1 and Level 2 scaffolds, we found that the top 1000 Level 1 scaffolds can represent most molecules in the three datasets, which demonstrates that the Level 1 scaffolds can serve as typical scaffolds to characterize the core structures present in the studied molecules in MDDR, ACD and TCMCD.

The similarity analysis for the scaffolds present in the studied datasets show that there are structural overlaps of scaffolds between MDDR and TCMCD, but TCMCD still contains many novel ring systems that cannot be found in MDDR. Finally, the Level 1 scaffolds for each dataset were clustered and visualized by Tree Maps. The results indicate that the distributions of the Level 1 scaffolds of MDDR are sparser than those of ACD and TCMCD, confirming the higher structural diversity of the scaffolds in MDDR. In addition, some Level 1 scaffolds with the highest frequencies of MDDR are similar to those of TCMCD, indicating that some ring substructures extracted from TCMCD may be served as valuable substructure resource for drug discovery.

## Competing interests

The authors declare that they have no competing interests.

## Authors’ contributions

ST wrote the paper, implemented the methods and conducted the analysis with assistance from YL, JW, XX, LX, XW, LC; TH contributed to the paper and provided guidance. All authors read and approved the final manuscript.

## Supplementary Material

Additional file 1 **The protocol to preprocess the three datasets. Figure S1:** The 20 most frequently occurring Level 1 scaffolds and their frequencies in (**a**) MDDR; (**b**) ACD and (**c**) TCMCD. Click here for file
