# Supplementary material for: Drug-likeness analysis of traditional Chinese medicines: 2. Characterization of scaffold architectures for drug-like compounds, non-drug-like compounds, and natural compounds from traditional Chinese medicines
Source: J Cheminform. 2013 Jan 21;5:5. doi: 10.1186/1758-2946-5-5 (PMC3561156; doi:10.1186/1758-2946-5-5)
Supplement: Additional file 1 — The protocol to preprocess the three datasets. Figure S1: The 20 most frequently occurring Level 1 scaffolds and their frequencies in (a) MDDR; (b) ACD and (c) TCMCD. [file 1758-2946-5-5-S1.doc]

**Supporting Materials**

**The protocol to preprocess the three datasets**. All the molecules in these three databases were minimized in MOE^1^ by using molecular mechanics (MM) with the MMFF94 force field.^2^ The three databases were preprocessed using the following protocol^3-6^: (1). Molecules were examined for bad valence states, and molecules containing one or more atoms with bad valence states were removed; (2). The salt fragments in the input molecules were identified and removed; (3). The molecules with atoms other than C, H, O, N, P, S, F, Cl, Br and I were removed; (4). The solvent molecules in the input molecules were identified and removed; (5). The input molecules with multiple organic parts were identified and the largest connected structural fragment in each input molecule was reserved; (6). Duplicates were removed in each individual database; (7). Identical compounds found in both ACD and MDDR databases were removed from ACD. For MDDR, antineoplastic drugs were removed because they are often highly cytotoxic and are likely to react with protein targets. In addition, the compounds (adsorption promoters, anesthetics, diagnostic agents (isotope), diagnostics for AIDS, diagnostics for cancer, drug delivery systems, magnetic resonance imaging agents, sweeteners, and dental agents) without therapeutic activity were eliminated from MDDR. As a result, we got 2,175,382 molecules from ACD, 142,747 molecules from MDDR and 63,759 molecules from TCMCD for the following analysis.

**References**


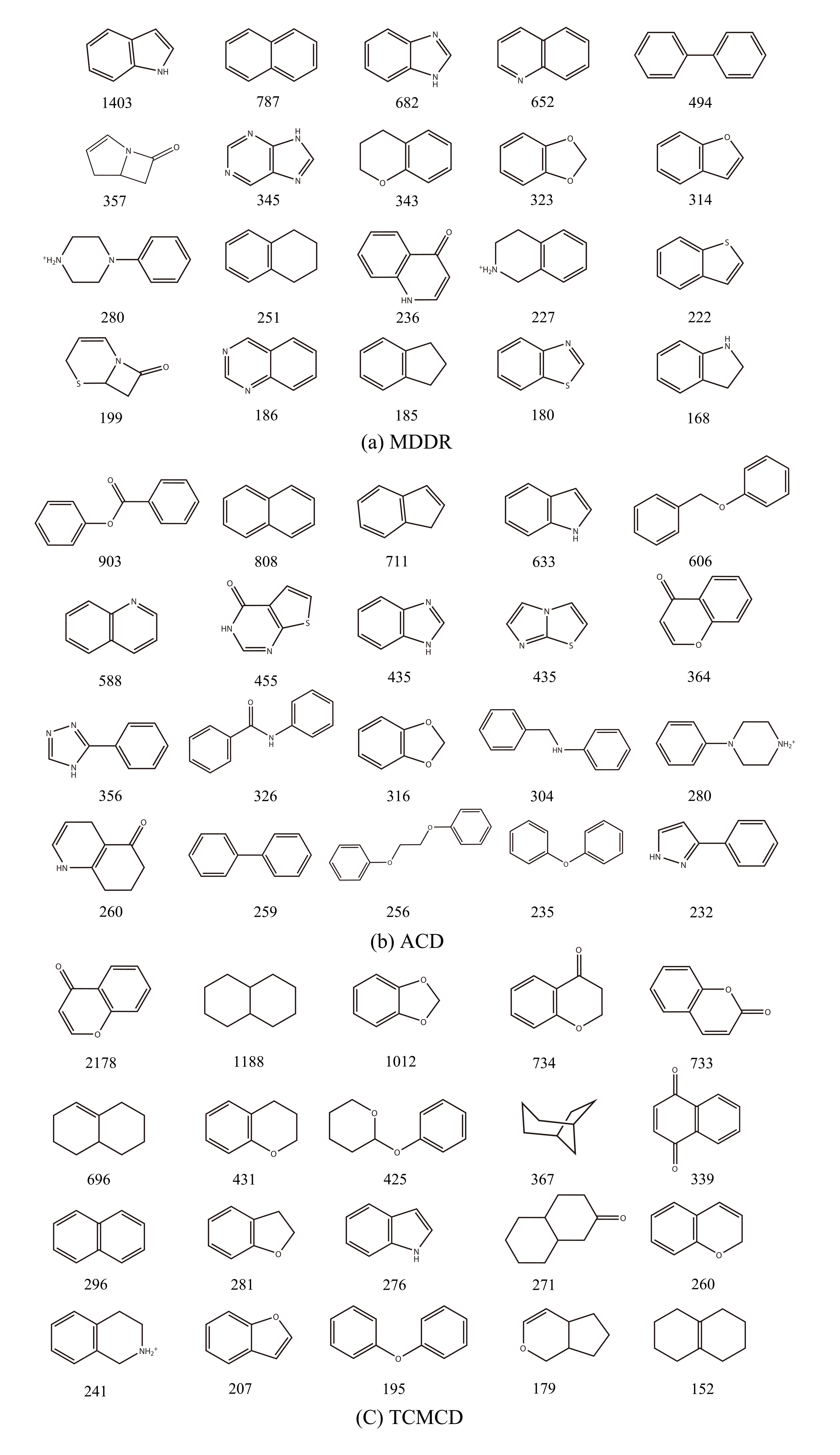


Figure S1
